# Parents’ experiences with sleep problems in children aged 1–3 years: a qualitative study from a health promotion perspective

**DOI:** 10.1080/17482631.2018.1527605

**Published:** 2018-10-05

**Authors:** Gro Sviggum, Ragnhild Sollesnes, Eva Langeland

**Affiliations:** Department of Health and Caring Sciences, Faculty of Health- and Social Sciences, Western Norway University of Applied Sciences, Bergen, Norway

**Keywords:** Sleep problems, small children, parents’ perspective, health promotion, qualitative content analysis

## Abstract

**Purpose**: The aim of this study was to explore the experiences of parents whose children aged 1–3 years have sleep problems, from a health promotion perspective.

**Methods**: This was a qualitative study, based on semi-structured interviews with 12 mothers in Norway. The material was analysed by qualitative content analysis.

**Results**: Parents experienced problems with getting their children to bed, getting them to fall asleep, and episodes of awakening at night. Parents expressed that it was time-consuming and difficult to teach their children sleep regulation. Parents handled the sleep problems through the following coping strategies: acknowledging challenges, clarifying one’s self-understanding, implementing change measures, and safeguarding family well-being. These coping strategies resulted in this main theme: the health-promoting regulation of interactions, including parents’ strengthening of sleep regulation in their small children and the safeguarding of well-being in the family.

**Conclusions**: Early, individually customized guidance for parents, with a focus on revealing and acknowledging their experiences with sleep problems in children, is essential for parents to find opportunities to cope with such challenges. Appropriate goals seem to be important for them to succeed in strengthening sleep regulation in their children in a more satisfactory way.

## Introduction

Good sleep is important for health and development in children (Hysing, Sivertsen, Garthus-Niegel, & Eberhard-Gran, 2016; Mindell & Owens, 2010). However, studies in Western countries show that parents are reporting sleep problems in 10–30% of children aged 1–3 years (Byars, Yolton, Rausch, Lanphear, & Beebe, 2012; Mindell & Owens, 2010), a problem that persists in many children (Byars et al., 2012). Reduced well-being in families (Eckerberg, 2004) and poor health and development in children such as tiredness and behavioural problems are common consequences (Hall, Scher, Zaidman-Zait, Espezel, & Warnock, 2011; Hysing et al., 2016; Mindell & Owens, 2010).

Sleep problems in small children are normally experienced by parents as unsatisfactory sleep habits in their children (Bharti, Mehta, & Malhi, 2013; Sadeh, Mindell, & Rivera, 2011). They often appear as difficulty getting children to bed in the evening, children taking a long time to fall asleep, and frequent episodes of awakening at night. It is known that children who develop sleep regulation, through “self-soothing” skills before falling asleep, often sleep in a more satisfactory way and have fewer episodes of awakening at night than do other children (Owens, Chervin, & Hoppin, 2017; Sadeh, Mindell, Luedtke, & Wiegand, 2009). Behavioural sleep problems in small children often involve the parents attempting to control sleep regulation in their children—such as by rocking them—or children becoming used to undesirable sleep-associated stimuli such as falling asleep with a bottle, so that they do not learn self-soothing before falling asleep. This can lead children to expect the same sleep-associated stimuli when they wake up at night, as they did when they fell asleep in the evening (Owens et al., 2017).

Behavioural approaches are often vital to promoting sleep regulation in small children (Mindell, Kuhn, Lewin, Meltzer, & Sadeh, 2006; Mindell & Owens, 2010). With such tactics, the best effect is normally achieved by using behavioural modification methods with support from health professionals (Mindell et al., 2006). This can promote the health of mothers (Hiscock, Bayer, Hampton, Ukoumunne, & Wake, 2008) and children, and well-being in their families (Eckerberg, 2004; Mindell et al., 2006). Behavioural modification methods describe, in a general way, how undesirable sleep-associated stimuli and the physical presence of the parents can be reduced, so that children become accustomed to falling asleep without these kinds of stimuli (Mindell & Owens, 2010). Different levels of behavioural modification methods involve parents gradually reducing sleep-associated stimuli, for example by increasing the period between each visit to the child’s bedroom (Sørensen, 2009).

In Norway, child health clinics offer guidance to parents concerning sleep and sleep habits in children aged 0–5 years. In these clinics, public health nurses and child health psychologists use research-based knowledge (Directorate of Health, 2017). However, as far as we know, there has been no research about how parents cope with sleep problems in small children from a subjective perspective. Therefore, the aim of this study from a health promotion perspective was to explore parents’ experiences of coping with sleep problems in small children aged 1–3 years.

## Theoretical framework

The salutogenic model (Antonovsky, 1979) was consulted as a strong health promotion theory (Antonovsky, 1996). In this model, health is seen as a resource on a continuum with different levels (Antonovsky, 1996). Health promotion measures focus on a sense of coherence, which is an expression of the individual’s ability to find and mobilize resistance resources to cope with challenges. One’s sense of coherence contributes to one’s health, and describes to what extent one has confidence that one’s own life experiences are comprehensible, manageable, and meaningful. These dimensions have to be seen in a specific context to make sense (Antonovsky, 1987). The goal in the salutogenic model is to promote a positive interaction between sense of coherence and resistance resources, to appropriately experience challenges in life, which contributes to a positive health pattern (Langeland & Vinje, 2013). Parents might be a crucial resistance resource for their children’s development of their sense of coherence (Antonovsky, 1987), and Stern’s development model (Stern, 2003) gives in-depth knowledge on the mutual interactions between parents and children, and the significance of parents’ role in developing memory and self-regulating behaviour in their children. Therefore, parents’ subjective experience of the feelings of their children has importance for how parents react and act in relation to their children. Further, this has importance for how children perceive and interpret parents’ subjective state of feeling, as something that has to do with the children themselves or the world in general. In this way, it is assumed that children develop self-esteem and a feeling of togetherness with their parents. This is important for children’s self-regulation and coping skills (Stern, 2003).

## Method

### Research design

The study had a qualitative research design with semi-structured interviews. Through this approach, our aim was to understand parents’ experiences rather than to explain them (Kvale & Brinkmann, 2009).

### Sampling

Twelve mothers of 12 children with sleeping problems volunteered to participate in the study. The participants were recruited from public health nurses and child health psychologists in service at child health clinics. To be included, prospective participants needed to be parents who had experienced sleep problems in their own child aged 1–3 years during the past 3 years. The problems should have had a duration of at least 3 months. Table I shows the socio-demographic characteristics of the participants.

**Table I.** Socio-demographic characteristics of the mothers (*n* = 12).

		*n*
Age (y)	20–29	5
	30–39	5
	40–45	2
Marital status	Married	5
	Cohabitating	6
	Single	1
Education	University or college:	
	> 4 years	1
	University or college:	
	< 4 years	8
	Comprehensive school/high school	2
	Not stated	1
Working situation	In a job	5
	Student	2
	Working at home	1
	Maternity	3
	Not stated	1
Number of children	One child	4
	Two or three children	8
Child’s age at the time of sleep problems	1–2 years	5
	2–3 years	7

### Data collection

The first author conducted the semi-structured interviews with them from November 2014 to May 2015. The interview guide was slightly adjusted after two pilot interviews. Open questions were asked at the beginning of the interviews, such as: “*Can you describe what you experience/experienced as sleep problems in your child?*” In the last part of the interview, clarifying questions were asked to verify that the participant had been understood correctly by the interviewers. Parents’ coping strategies were evaluated by letting them express what they felt had helped so far, what was helping in the current situation, and what they felt could help in the future. It was important for the interviewer to be aware of her own preconceptions and to be open-minded, in order to comprehend the essence of the mothers’ experiences. The interviews lasted 50–60 minutes and were conducted at child health clinics or in the parents’ homes, often after the children had gone to sleep. Each participant was interviewed once. The interviews were recorded.

### Analysis

Our purpose was to organize and find structure and meaning in the data (Polit & Beck, 2017). This work started by aiming to have a good overview and to write a report after each interview, and to make verbatim transcriptions from the recordings. The material was then analysed, following the approach of Graneheim and Lundman (2004). In the qualitative content analysis, words, sentences or paragraphs were collected in meaning units in each interview. The text in the meaning units was thereafter shortened down and coded. Then the preliminary meaning units were analysed across participants and it was developed final categories and an overall theme that safeguarded the participants’ experiences. The three authors discussed the analysis process and agreed upon the theme and categories. Table II shows an example of the qualitative content analysis.

**Table II.** Example of the qualitative content analysis.

Meaning unit	Codes	Sub-categories	Category	Theme
“*Routines are good for all of us. It can easily get hard if we do not follow them*.” “*And then we go and brush the teeth and go to bed and read a book and sing two songs. It is one book and two songs*.” “*We have a snuggle time together in the bathroom, where we look at a book before going to bed*.”	Sleep routines are vital for well-being in the family. Sleep routines are important for children’s understanding of going to bed and falling asleep. Calm togetherness with children and sleep routines are important to promote children’s understanding of going to bed and falling asleep.	Safeguard sleep routines. Safeguard inner peace.	Safeguard family well-being.	Health-promoting regulation of interactions, including parents’ strengthening of sleep regulation in their small children and safeguarding of well-being in the family.

### Ethical considerations

The Regional Committee for Medical and Health Research Ethics for Western Norway approved the study in June 2014 (REK Vest Number 2014/1055). The participants were given written information about the study and their rights, where it was specifically pointed out that they could withdraw from the study at any time without needing to give an explanation. They all gave written consent to participate in the study. The data were anonymized and stored confidentially.

## Results

**Figure 1.**
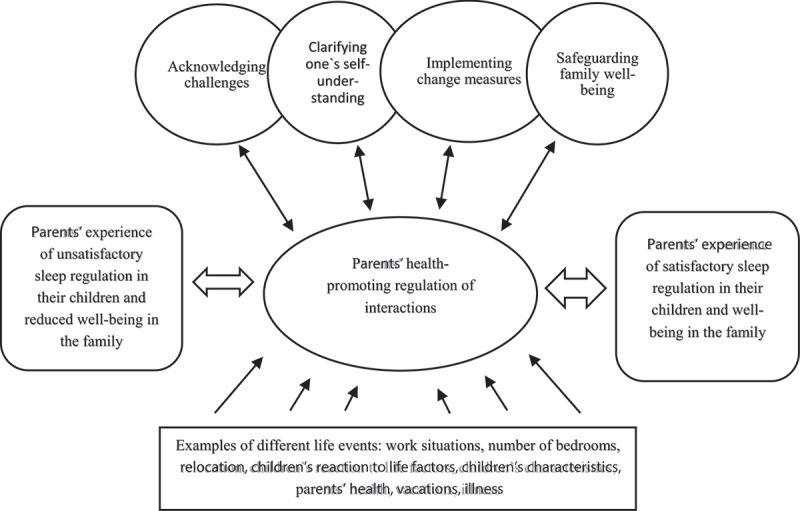
A health promotion approach in regulating interactions, including parents’ strengthening of sleep regulation in their small children, and safeguarding of well-being in the family.

Parents reported that they experienced sleep problems in their children at the time of the interviews. Some parents reported that the sleep problems recently had been solved. The parents experienced problems with their children when putting them to bed, trying to get them to fall asleep, and during episodes of awakening at night. Through analysis, four coping categories were identified: acknowledging challenges, clarifying one’s self-understanding, implementing change measures, and safeguarding family well-being. These categories led to the following theme: health-promoting regulation of interactions, including parents’ strengthening of sleep regulation in their small children and safeguarding of well-being in the family. The parents considered sleep regulation in the children as a developmental goal to be achieved at their own pace. This meant that the children had to learn how to calm down by themselves in their own bed before falling asleep in the evening and when waking up during the night after a while in their own room, without sleep stimulation help from the parents such as getting a drink. The parents often experienced this as an unsatisfactorily long “back and forth” process, caused by different occurrences and situations that influenced the chain of events, and often the well-being of the family. Nevertheless, the parents’ strengthened the children’s sleep regulation by adjusting their interactions through customized use of the four coping categories. Figure 1 shows parents’ experiences, where the broad double-ended arrows describe the “back and forth” process.

### Parents’ health-promoting regulation of interactions

#### Acknowledging challenges

##### Acknowledging unrest

Mothers reported that the children were often uneasy when parents were not physically nearby and able to help them calm down, especially during the process of falling asleep in the evening or when wakening up at night. As one mother described: “*He wakes up at least three times, but it can be up to five. Then he whines and wants me to take him up and breastfeed him*.” Parents often became accustomed to much unrest, as another mother described: “*We have had it like that for several years. We have got used to being woken up a number of times each night*.” The sleep problems often led to parents being tired and worn out, with little energy left to meet other expectations in their everyday life. Thus, one mother said: “*I was very tired, very tired. I remember I was dreading his brother starting school*.” Limited energy was likely to amplify difficult feelings, which promoted the parents’ acknowledgement of having challenges. Another described: “*When you have had a hard night and you still have to get up at half past four, one of those nights we realized that we needed to start being nicer to each other*.” Increased levels of tension often led to better awareness in the parents.

##### Clarifying challenges

Parents often questioned why the children were uneasy, what they were doing wrong as parents, and how they could make things work better. Unanswered questions made it difficult to change sleep habits. Parents struggled with regulating children’s sleep during the daytime to ensure that the children would have a suitable level of tiredness at a desirable time for falling asleep in the evening. Varying level of tiredness often made it difficult for the parents to know how much sleep regulation they could expect from the children. Parents often questioned whether major, abrupt changes in sleep habits could be negative, as this mother described: “*I have tried ‘crying cures’ and to get her used to not being breastfed during the night, but it has worked very badly. I am not sure if I can go through it again, because if it takes a long time, it affects both of us, and maybe it can also affect our relationship*.” A significant challenge for parents was making their children understand new sleep habits. A mother described it as follows: “*The most important thing was that she felt we had not rejected her, but had made her understand that it was for her own good*.” The parents often largely accepted living with the sleep problems. However, on the other hand, this was especially challenging during life changes, as expressed by this mother: “*I then started to feel the need to have nights of uninterrupted sleep, since I was going to work during the daytime*.” Parents’ confirmation of challenges led to a need to understand how they could be solved.

#### Clarifying one’s self-understanding

##### Acquiring understanding

Parents often searched for knowledge about the challenges they were facing. One mother said: “*We have gathered information that has seemed appropriate for us*.” New knowledge contributed to parents being dissociated from some advice or from making useful discoveries. Another response was: “*After we were recommended to teach him how to fall asleep without stimulation, we have got a totally different world*.” It was especially important for parents to find out how they could teach their children sleep regulation in a safe way. Therefore, the parents considered the children’s need for care by observing their feelings and behaviour, especially during bedtime and episodes of awakening at night, but also in general during the daytime, such as considering how the children reacted when they were put into their own bed. An important question was how much physical distance the children and parents could achieve, without it becoming too difficult emotionally. One mother said: “*He had been crying, not because he had lost his pacifier or such a thing, but because he had been upset. So, we had to come*.” Another said: “*It was a good experience when we started to hear that she sounded angry. So, now we are able to let her lie for a while*.” Parents also considered values in the parent role in general and relative to their own life situation. One mother expressed it as follows: “*He gets comfort when he needs it and obtains contact with us when he shouts. To fight one’s own children to make them sleep tears me apart*.” Another expressed it in this way: “*If we should have done anything differently, it must have been to let him cry more, gradually, as he became more secure. As a matter of fact, we do everything we can for them not to cry, but maybe it is not such a big deal*.” Sufficient knowledge was important to be able to acknowledge the parents’ own understanding.

##### Acknowledging one’s own understanding

Parents often assessed whether their life situation was sufficiently suitable to be able to succeed in strengthening the children’s sleep regulation more effectively. It was important to understand their own perspective well enough to acknowledge how much they could expect from their children’s sleep regulation. Individual life factors, such as job relocation, influenced parents’ considerations a great deal. General factors such as practical aids, cooperation with one’s spouse or cohabitant, relief help, and extra energy also had vital importance. It was important to learn from their own experiences. Some mothers expressed this as follows: “*We are in a kind of situation where we do not know how hard we would like to work on it [sleep regulation] for the time being*,” and “*It is important to listen to oneself*.” Because of contradicting experiences, it was sometimes difficult for parents to acknowledge their own status emotionally, as this mother said: “*I love to get into bed with her. So, for me, it is more like, I have not had a strong enough wish to change things. But I think she could gain from coping with falling asleep by herself*.” Thus, parents clarifying their own status could lead to small or big changes in their children’s sleep habits.

#### Implementing change measures

##### Changing from the usual sleep habits towards new ones

During their first year, the children had often slept fully or partially in their parents’ beds, with gradually increasing time in their own beds, which had often been placed in the parents’ bedrooms. The mothers had normally breastfed or performed other sleep-promoting measures to help the children fall asleep. To allow the children to sleep in near proximity had often been necessary, to be able to calm them quickly during the night. This had also been important for promoting affiliation and safety, especially during periods with disturbing life factors, for example illness. Different sleep-promoting habits had often persisted, such as breastfeeding. However, for almost all the parents it had become necessary to strengthen the children’s sleep regulation more effectively in the children’s own rooms, to avoid disturbing one another by sleeping in the same room, among other reasons. Parents often experienced this adjustment as difficult, and found that it took an unsatisfactorily long time to teach the children sleep regulation in their own bedrooms. One mother expressed it like this: “*If it had worked after three days, I would have preferred it; not to go through all of this*.” Another mother described a ‘*back and forth*’ process, as follows: “*I remember she seemed a bit calmer then. But now it is back again, so it is not that easy to calm her*.” Some parents had tried to teach the children sleep regulation over a shorter time, with different levels of behavioural modification methods. However, allowing the transition to take place over a long time was the best option for most of them, because the children often became insecure and it was too emotionally difficult to make major changes in a short time. Both children and parents could cope with smaller changes. One mother expressed it as follows: “*We cannot make her sleep on her own. But we sleep in the room next door, so the next project will be us talking to her from our bedroom without going into hers*.” Some parents had experienced markedly better sleep regulation and all-night sleep in their children, and increased well-being in the family, after 3–7 days by using specific behavioural modification methods. For these parents, it had been necessary to completely cease inexpedient sleep habits, such as caressing the children to make them fall asleep. Further on, it had been important to give more indirect emotional support to the children, such as verbal encouragement, which then had been gradually reduced. One mother described it as follows: “*We looked at the watch when we were about to go in to him. It was comforting to know that he was fine and not hungry. So, I thought, we are here, let him know that we are here, and I thought that it should not create insecurity in him*.”

In general, confidence-building measures were important when teaching sleep regulation; for example, teaching the children to fall asleep with a teddy bear. Safety and well-being also needed to be promoted during the daytime to strengthen the children’s sense of inner peace.

#### Safeguarding family well-being

##### Safeguarding sleep routines

The parents were focused on the idea that both they and the children needed to follow satisfactory routines and sleep habits, both old and new. One mother pinpointed the importance of this: “*Routines are the pathway to all good outcomes for us*.” Routines promoted good circadian rhythms and prevented unrest. Sleep routines in the evening were especially important. Parents often acknowledged their own importance in safeguarding children’s inner peace before falling asleep, and were therefore likely to downgrade other tasks, such as visits to friends. Setting limits for themselves, the children, or others could be necessary to shield their children from adverse stimuli, and to calm down their activity level 1–2 hours prior to bedtime. Fixed bedtimes, eventually a morning nap of specific duration, and avoiding an afternoon nap all promoted good circadian rhythms in the children and prevented unrest. Regular meals and time for play and other activity during the day contributed to the children being tired and satisfied in the evening.

Safeguarding inner peace: The parents talked about the importance of seeing their children and showing them understanding. Some expressed that it was also important to understand one another, and themselves. This reflected a wish that everyone could live a meaningful life, as a safeguarding of identity to strengthen inner peace and development, which was believed to help promote satisfactory sleep in the children. For parents, this could be about well-being in a study situation, job, or working at home, or being together with spouse/cohabitant or other close relations, or being able to spend time alone. It was important to find a balance between coping with expectations and having free time. One parent described it in this way: “*We were very happy, we had a good time, and it was nice. It gave us an ‘energy boost’ to keep on succeeding*.” To have time off from expectations gave parents opportunities to deal with their emotions and to get confirmation of being one of many who had experienced similar sleep problems in small children. The parents also arranged for good moments with the children, with quality time characterized by love, gratitude, and well-being. Such moments with enough time and peace were important to promote development in the family, and to safeguard the affiliation between parents and children. Parents often expressed that it was important to create inner peace in the children by explaining current and planned activities, and by making children’s daily routines understandable and predictable. One mother described it as follows: “*She gets to watch some children’s TV while she eats her evening meal. And then we brush her teeth before we go to bed and read a book and sing two songs. Then we leave the room. She acknowledges that only one book is allowed*.” In general, well-being promoted inner peace in the parents, which was perceived as helping to strengthen sleep regulation and all-night sleep in their children.

## Discussion

The parents in this study coped with sleep problems in their small children by alternating between acknowledging challenges, clarifying one’s self-understanding, implementing change measures, and safeguarding family well-being. This led to the following theme: the health-promoting regulation of interactions.

### Creating a sense of order

Our study showed that parents had experienced difficulties in getting their children to go to bed and fall asleep, and episodes of awakening at night, as the most common sleep problems. This has also been shown in other studies (Byars et al., 2012; Mindell & Owens, 2010). The parents performed considerable demanding care work to help their children get to sleep, while they themselves often experienced reduced or disturbed sleep, leaving them tired and exhausted. This was also highlighted in other studies (Cooklin, Giallo, & Rose, 2012; Giallo, Rose, Cooklin, & McCormack, 2013; Giallo, Rose, & Vittorino, 2011), and is something that can be seen in the context of the experience of fatigue among parents, including after the first few years of a child’s life (Cooklin et al., 2012). It is known that sleep problems in small children can be emotionally difficult for mothers (Hiscock et al., 2008). This was also shown in our study, apparently because the mothers often felt that the children were having a difficult time, and therefore stretched themselves emotionally and physically to safeguard the children’s need for closeness, peace, and sleep. In addition, mothers stretched themselves to safeguard the well-being of the family, and often suppressed their own need for relaxation and sleep. Therefore, it can be important to support parents in safeguarding their own care to a larger degree, as also recommended by Giallo et al. (2013). In our study, most of the families had largely adjusted to the sleep problems. This was also found by Tse and Hall (2008). Because families are likely to live with sleep problems in small children over a period of time, it can be important to help parents to acknowledge their own strain and overload to a larger degree. Further, the study showed that parents often experienced many challenges, which were often unclear and difficult to resolve. Parents’ gradual recognition of their own experiences promoted a sense of order and led to better strategies for coping with stress using different resources. This finding is in agreement with the salutogenic theory, in which coping is promoted by a better understanding of one’s own situation and then the use of different available and conscious resistance resources (Antonovsky, 1979).

Our study showed that it was important for parents to acquire knowledge customized to the family’s life situation. Customized knowledge was a key to strengthening parents’ expectations and to safeguarding the children’s and their own safety when the children needed to learn sleep regulation. Safety as a form of inner peace is about how people understand and cope with challenges meaningfully (Antonovsky, 1987). Coping with life events influenced how effective parents expected to strengthen the children’s sleep regulation. Parents’ perception of children’s emotional reactions to different life events, such as trying to understand the world through a child’s eyes, was important for how they considered the children’s inner peace at bedtime and during subsequent episodes of awakening at night. Parents’ empathy with their children’s experience of the world is also emphasized in Stern’s theory (Stern, 2003). Therefore, for guidance, it is important for parents to express their perception of their children’s emotional state at bedtime and during episodes of awakening at night. For the parents, it was important to struggle to make the children understand new sleep habits. Understanding is a key dimension in the sense of coherence concept, and sense of coherence develops through coherent life experiences (Antonovsky, 1987). Therefore, it can be important to guide parents in how they can strengthen the individual child’s sense of coherence in conjunction with the children’s sleep regulation. This is about helping parents to understand the individual child’s feelings during the development of sleep regulation and to find strategies that can promote safety. Parents’ perception of often unpredictable events means that their expectations when teaching children sleep regulation often switched between doubt and hope. Life events meant that the children’s development often appeared as a ‘*back and forth*’ process. A similar process has been described indirectly as a relapse, triggered by disruptions of routines, such as teething (Tse & Hall, 2008). Therefore, a family’s self-perception is important to communicate when guiding parents. This supports the idea that sleep problems in small children should be seen in the context of the situation of the individual child and family (Mindell & Owens, 2010; Sadeh, Tikotzky, & Scher, 2010). The theory of salutogenesis sheds light on this by emphasizing that a positive interaction between sense of coherence and resistance resources is important for health promotion (Langeland & Vinje, 2013). Moreover, the study also showed that it was important for parents to acknowledge their own self-understanding sufficiently before being able to implement any change measures. Such a recognition could take some time, and is likely to be difficult, especially in a challenging life situation. Langeland and Vinje (2013) stated that it is important to promote self-respect in the individual by accepting stressful experiences as normal. The crucial point is to prevent stress and tension from persisting for a long time by gradually develop a sense of order out of rather stressful experiences. Thus, good guidance to parents should comprise a balance between listening empathically to problems and giving attention to the strengths and resources of the parent being guided. In this way, stress and tension can be transformed into coping strategies (Langeland, Gjengedal, & Vinje, 2016; Langeland & Vinje, 2013).

### Promoting coping and well-being

We found that the parents who had benefited from specific behavioural modification methods did not express many concerns about whether the methods were customized to the life situation of their own family. On the other hand, they showed clear expectations that the chosen method would help. However, doubt regarding whether their children could develop sleep regulation in a short period of time was normal among the parents. Several were worried that the methods could have negative consequences, for example on their children’s mental health. In a study with interviews of 14 parents (Tse & Hall, 2008), similarly varying experiences with a specific behavioural modification method were revealed. In our study, parents who strengthened their children’s sleep regulation over a period stated that the children’s emotional state at bedtime and during episodes of awakening at night was normally an expression of their need for contact with their parents. This was often seen in the context of a challenging life situation. On the other hand, parents who had benefited from specific behavioural modification methods did not feel that their children had been insecure at bedtime or during episodes of awakening at night, either before or during use of the methods. Therefore, a life situation with sufficient inner peace in the parents and children appears to be essential for parents to be able to strengthen sleep regulation significantly in their small children in a satisfactory way over a short period. Appropriate coping goals are assumed to be important for parents to succeed in strengthening their children’s development of sleep regulation.

A state of health includes confidence that the world is experienced as understandable, structured, and predictable (Antonovsky, 1987). In this study, parents promoted the children’s confidence in the world by facilitating understandable, structured, and predictable sleep habits and circadian rhythms, and meaningful togetherness. These measures promoted inner peace in the children. The parents often fulfilled their own expectations to promote a trustworthy world for the children, where safety was important. On the other hand, in general the parents rarely expressed the importance of promoting predictability in their own bedtime routines and sleep habits. The mothers’ general need for well-being was also marginalized, relative to safeguarding the well-being of their children and the family. Therefore, it can be relevant to guide parents in how to safeguard their own needs, while at the same time taking into account their need to safeguard the children and the family. Ways to recover one’s strength are important in promoting good coping, meaning and well-being in life and in avoiding parental overload (Antonovsky, 1987).

## Strengths and limitations

As far as we know, this has been the first study to explore parents’ coping with sleep problems in small children from a health promotion perspective. Therefore, the study contributes to increased understanding of an issue that concerns many parents of small children. It has been important to describe and explore parents’ experiences respectfully to promote trustworthy knowledge. However, we need more studies about parents’ coping with sleep problems in their small children in which fathers also are included.

## Conclusions

The study has highlighted that it is essential to offer early individual guidance to parents struggling with sleep problems in their children. An important health-promoting factor is parents’ acknowledgment of their own life situation and safeguarding of their own needs. A clarification of the self-understanding of the family regarding life events that influence the children’s development of sleep regulation is another key to helping parents in finding appropriate coping strategies, aimed at promoting realistic expectations and strengthening their children’s development of sleep regulation in a more satisfactory way.
